# Evaluation of the Impact of Chemical Mutagens on the Phenological and Biochemical Characteristics of Two Varieties of Soybean (*Glycine max* L.)

**DOI:** 10.3390/life14070909

**Published:** 2024-07-22

**Authors:** Anas Hamisu, Bhupendra Koul, Ananta Prasad Arukha, Saleh Al Nadhari, Muhammad Fazle Rabbee

**Affiliations:** 1 Department of Botany, School of Bioengineering and Biosciences, Lovely Professional University, Phagwara 144411, Punjab, India; anashamisu213@gmail.com; 2 Department of Nephrology and Hypertension, Mayo Medical Sciences, Rochester, MN 55902, USA; ananta.arukhaa@gmail.com; 3 Deanship of Scientific Research, King Saud University, Riyadh 11451, Saudi Arabia; salnadhari@ksu.edu.sa; 4 Department of Biotechnology, Yeungnam University, Gyeongsan 38541, Republic of Korea

**Keywords:** mutagens, *Glycine max*, crop improvement, induced mutation

## Abstract

Mutagenic effectiveness and efficiency are the most important factors determining the success of mutation breeding, a coherent tool for quickly enhancing diversity in crops. This study was carried out at Lovely Professional University’s agricultural research farm in Punjab, India, during the year 2023. The experimental design followed a randomized complete block design (RCBD) with three replications. The experiment aimed to assess the effect of three chemical mutagens, sodium azide (SA), ethyl methyl sulphonates (EMSs), and methyl methane sulfonate (MMS), at three different concentrations (0.2%, 0.4%, and 0.6%), in SL958 and SL744 soybean varieties to select the mutant exhibiting the highest yield. The data were collected and analysed using a two-way ANOVA test through SPSS software (version 22), and the means were separated using Duncan’s multiple range test (DMRT) at the 5% level of significance. Between the two varieties, the highest seed germination percentage (76.0% seedlings/plot) was recorded in SL958 (0.4% SA), while the lowest (30.33% seedlings/plot) was observed in 0.6% MMS as compared to the control (53% and 76% in SL744 and SL958 at 10 days after sowing, respectively). Several weeks after sowing, the average plant height was observed to be higher (37.84 ± 1.32 cm) in SL958 (0.4% SA) and lower (20.58 ± 0.30 cm) in SL744 (0.6% SA), as compared to the controls (SL958: 26.09 ± 0.62 cm and SL744: 27.48 ± 0.74 cm). The average leaf count was the highest (234.33 ± 3.09 tetrafoliate leaves/plant) in SL958 (0.4% SA) while it was the lowest (87 leaves/plant) in 0.6% MMS as compared to the control (SL744 180.00 ± 1.63 and SL958 160.73 ± 1.05). The highest total leaf areas recorded in the SL958 and SL744 M1plants were 3625.8 ± 1.43 cm^2^ and 2311.03 ± 3.65 cm^2^, respectively. Seeds of the SL958 variety treated with 0.4% SA resulted in the development of tetrafoliate leaves with a broad leaf base and the maximum yield (277.55 ± 1.37 pods/plant) compared to the narrow pentafoliate leaves obtained through the treatment with EMS. Meanwhile, in the SL744 variety, the same treatment led to tetrafoliate leaves with a comparatively lower yield of 206.54 ± 23.47 pods/plant as compared to the control (SL744 164.33 ± 8.58 and SL958 229.86 ± 0.96). The highest protein content (47.04 ± 0.87% TSP) was recorded in the SL958 (0.4% SA) M2 seeds followed by a content of 46.14 ± 0.64% TSP in the SL744 (0.4% SA) M2 seeds, whereas the lowest content (38.13 ± 0.81% TSP) was found in SL958 (0.6% MMS). Similar observations were recorded for the lipid and fibre content. The 0.4% SA treatment in SL958 proved to be efficient in generating the highest leaf area (tetrafoliate leaves) and a reasonable yield of M1 (the first generation after mutation) plants.

## 1. Introduction

The term plant mutation is used to describe a sudden heritable change in the genetic material of a plant at the gene and chromosome level. Mutations are the tools used to study the nature and function of genes, which are responsible for quality traits (disease resistance, early maturation, semi-dwarfism, etc.) and quantity traits (increased seed productivity, seed weight, biomass, oil content, etc.) in crops, thus acting as means for the genetic improvement of economic crops [1,2]. Mutation breeding (variant breeding) is a technique for creating novel crop varieties by inducing genetic diversity in plants using chemical or physical techniques [1,2].

The advantages of mutation breeding are as follows: (i) the improvement of economic traits and quality characteristics in crops within a short period of time, (ii) traits can be transferred from a mutant variety to its offspring, (iii) the technique is simple and inexpensive, (iv) reliable, and (v) hazard-free and environment-friendly. Its disadvantages include the following: (i) it requires stringent screening for the mutant with the desired trait, (ii) there is a chance of unpredictable outcomes due to random genetic recombination, and (iii) its success relies on the desired qualities being present in the current gene pool [3,4].

There are two types of mutagens: (i) physical mutagens, including (a) ionizing (particulate: alpha, beta, fast, and thermal neutrons; non-particulate: X-rays and gamma rays) and (b) non-ionizing (UV-rays); and (ii) chemical mutagens, such as (a) alkylating agents: EMS (ethyl methane sulfonate), MMS (methyl methane sulfonate), and mustard gas; (b) deamination agents (nitrous acid); (c) base analogues (5-bromouracil and 2-aminopurine); (d) acridines (acriflavine and proflavin); and (e) other chemicals (hydroxylamine and sodium azide). Chemical mutagens are easy to handle, readily available, inexpensive, cost-effective, more efficient, and have a greater specificity than physical mutagens [5]. The genetic makeup of economic crops has been improved by mutation breeding along with a significant increase in crop production. The improvement of economic traits and quality characteristics for the improvement of crops within a short period can be achieved with the help of induced mutagenesis. Induced morphological mutants are useful for the development of improved varieties when they are used in cross-breeding programs [6].

Despite their benefits and drawbacks, mutagens have been used to create several better cultivars of many crops, including a wheat mutant (Sharbati Sonora) of an amber-coloured grain variety with a higher protein and lysine content developed through gamma irradiation [7]; a rice mutant of the TEMB-1 variety with a semi-dwarf phenotype (90–95 cm) that matures early (130–135 days), lodging-resistant, aromatic, and has a high yield (4.5–5 tons per ha) [8,9]; a barley mutant of the diamant variety with large kernels that matures early and has high yields [10]; a soybean mutant of the TGX-1954 variety that matures early, drought tolerant, disease resistant, and has a higher yield [11]; a chickpea mutant of the BGM-413 variety that is salinity tolerant, tastes good, can be used in cooking, matures early, has a high yield, and has a high protein content [12]; a cotton mutant of a virescent variety with early maturation, disease resistance, drought tolerance, and a higher yield capacity [13]; a sugar cane mutant of the Co0238 variety that has a high fibre content (13.05%) [14]; a tea mutant that is early sprouting, resistant to disease, and suitable for fine green tea developed through irradiation (Co60c) [15]; and a vegetable mutant of the *Capsicum annum* variety with a high yield, increased vitamin content, and disease resistance [16,17]. Thus, many features, including yield, lodging resistance, disease resistance, maturity, culm length, etc., have been developed through mutation breeding.

The soybean (*Glycine max* (L.) Merrill) is known as the “golden bean” and the “miracle crop” of the twenty-first century. It contains high-quality oil (20%), protein (40%), starch (36–40%), vitamins A, B, and D, 6–7% minerals (calcium, iron, manganese, phosphorus, copper, and thiamine), 8–10% fibre, and phytochemicals such as flavonoids, saponins, alkaloids, steroids, and tannins [18].

In India, soybean is also used in various food products, including tofu, soya sauce, simulated milk, and meat products, and the oil is frequently used for cooking [19]. Soybean meal is used as a supplement in livestock feed. Apart from this, it is used in industries for the production of biofuels and functional foods [20]. The industrial use of soybeans ranges from the production of yeasts and antibodies to the manufacture of soaps and disinfectants [21]. The top five soybean-producing nations worldwide are Brazil (120.7 MT), the United States (116.4 MT), Argentina (43.9 MT), China (20.3 MT), and India (13 MT). More than 90% of the world’s production comes from these countries [19]. India is the fifth largest soybean-producing country in the world. Madhya Pradesh, Rajasthan, and Maharashtra contribute 88% of the country’s total soybean production [20]. Soybean can play an important role in crop diversification in Punjab, provided it is able to compete with paddy, wheat, and cowpea in terms of economics. Yellow mosaic is a serious disease in the northwest’s plains, causing yield losses up to 80 percent. Thus, the development of high-yielding and yellow-mosaic-resistant varieties is the main breeding objective of the soybean breeding programme at Punjab Agricultural University, Ludhiana, India. The two most recent varieties, SL958 and SL744, have been released. These have medium levels of maturity, are non-shattering, have a light yellow grain with a grey hilum, and are resistant to the yellow mosaic virus [22,23].

In the 1970s and 1980s, as reported by the International Food Policy Research Institute, Punjab was among the highest producers of soybean with a net domestic product (NDP) of 5.1–6.1 MT on average compared to the national average of 3.4 for India [24]. Agriculture in Punjab is limited to wheat and rice, which has resulted in the excess mining of underground water resources for irrigation. Over the past 54 years, this has lowered the water table to the extent of 15 cm per year. Soybean production might serve as a supplement or as part of crop rotation, since it requires a lower amount of water.

This study assesses the impact of three chemical mutagens, sodium azide, ethyl methyl sulphonates, and methyl methane sulphonate, on the two aforementioned soybean varieties through field trials, with the aim of unveiling their potential to generate genetic variability and screening the improved soybeans with a higher yield potential.

## 2. Materials and Methods

### 2.1. Seed Procurement and Germination Test

Soybean seeds were obtained from Punjab University of Agriculture, Ludhiana, India (30.9041° N latitude and 75.8066° E longitude). Two soybean varieties (SL958 and SL744) were selected based on their characteristics, including their high yield, medium level of maturity, and soil preference. A germination test was conducted by broadcasting a hundred seeds of SL744 and SL 958 in two earthen pots filled with fertile organic soil. Following the placement of seeds, a light covering of soil was applied, and the pots were consistently watered. Germination percentage was closely observed over five days, with results indicating up to 95% germination. The cleared and manually ploughed site for seed production was laid out according to the experimental plot design (RCBD) measuring 8 × 60 m^2^, and seeds were sown accordingly.

### 2.2. Mutagens’ Procurement and Seed Treatments

Chemicals were obtained from Loba chemie Pvt. Ltd., Mumbai, India. The seeds were soaked in distilled water for 6 h and later on treated with ethyl methane sulphonate (EMS), methyl methane sulfonate (MMS), and sodium azide (SA) for 6 h. They were immersed at different concentrations (0.0%, 0.2%, 0.4%, 0.6%) of three chemicals, respectively. The 0.0% concentration was the control. Fifty mL phosphate buffer solution was added to each treatment so as to maintain the osmotic content of the cell at pH of 3. Another set of seeds were soaked in distilled water and phosphate buffer solution as a control. The procedure was periodically repeated at room temperature (25–30 °C). After the end of the treatments, seeds were extensively rinsed with running tap water approximately 8–10 times. Subsequently, both treated and untreated control seeds were sown in the field using a randomized complete block design with three replications to induce mutation generation [25].

### 2.3. Field Location and Experimental Design

**Figure 1:**
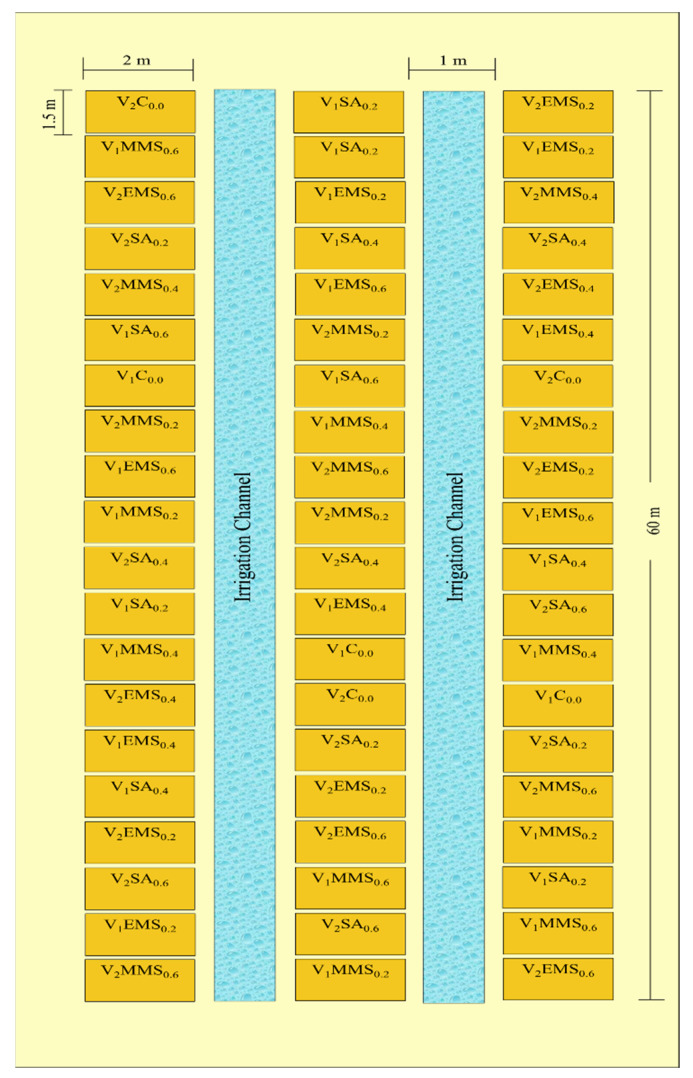
Field layout (8 × 60 m) and experimental design using RBD. V1: variety 1 (SL744), V2: variety 2 (SL958), C: control. Chemical_xx_ (xx = 0.2, 0.4, 0.6%), EMS: ethyl methane sulfonate, MMS: methyl methane sulfonate, SA: sodium azide.

The research was carried out at Lovely Professional University’s agricultural research farm (31.3192° N latitude and 75.5723° E longitude) during the year 2023. The area lies within the sub-tropical belt in Northwest India. Semi-arid deserts of Rajasthan lie towards the southern border. It is characterized by three major seasons: summer (April to June); rainy (July to September); and winter (October to March). The climate ranges from sub-humid (northeast) to semi-arid (southwest), with harsh winters and summers. The area is characterised by sandy loam soil. Temperature ranges from −4 °C to 47 °C. Average annual rainfall is 650 mm. The land area measuring 620 m^2^ was divided into 60 plots (3 columns of 20 plots each), each measuring 2 × 1.5 m^2^, with a spacing of 0.75 m between plots and 1 m gap (as irrigation channel) between replications (Figure 1). Each plot consisted of four ridges that were spaced 0.37 m apart, with the two innermost ridges serving as net plots. The rows’ alley was used for destructive and non-destructive sampling, while the border rows were used for discard sampling. The experimental design followed a randomized complete block design (RCBD) with three replications.

Before planting, the soil was ploughed, harrowed, and ridged to remove debris, weeds, and clods for proper aeration and moisture for crop growth. Seeds were sown with a spacing of 75 cm on each ridge immediately after the rain during third week of May 2023.

Three seeds were manually sown per hole and later thinned into two plants per stand at 2 weeks after sowing (WAS). They were harvested manually using hoe and sickle. Physiologically matured pods were collected and sun-dried to reduce the moisture content, and later, manual threshing was carried out to separate the seeds from the chaff.

### 2.4. Plant Growth Characteristics

Various plant growth characteristics (seed germination, plant height, leaf number, leaf area, leaf variation, nodule counts, days to 50% flowering, and days to 50% podding) were recorded for the control and the M1 plants of two soybean varieties (SL744 and SL958) at the different stages of their growth after sowing.

**Figure 2:**
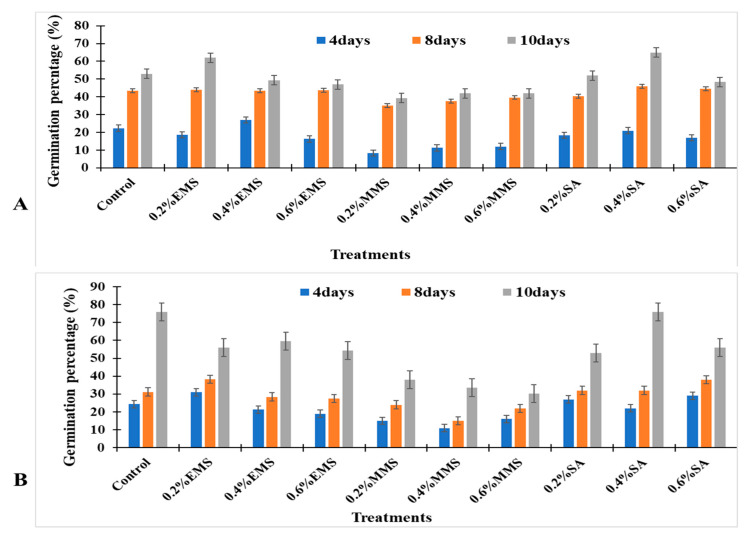
Effect of chemical mutagens on seed germination percentage (%). (**A**) Effect on SL744 soybean varieties. (**B**) Effect on SL958 soybean varieties. DAS = Days after sowing.

For calculation of germination percentage, 96 seeds were sown in each plot. After seed germination, the seedlings were counted, and seed germination percentages were recorded for control and test plots (as shown in Figure 2), which were compared statistically. The seed germination percentages were used to plot the graphs (*x* axis: chemical treatments; *y* axis: seed germination percentage) using MS Excel version 13. Plant height was taken at intervals of 4, 8, and 12 weeks after sowing. Five plants were randomly selected from each plot, and their heights were measured from base to apex using a measuring tape. The average heights of the M1 plants were compared with those of the respective controls.

The number of leaves and leaf area per plant were also recorded at intervals of 4, 8, and 12 weeks after sowing. Five plants were randomly selected from each plot for leaf count and leaf area, and then average values were recorded for control and M1 plants of both varieties, which were later compared statistically. The leaf variation between the two varieties in the test plots was also studied at the aforementioned intervals, and the data were compared with the respective controls. The leaf chlorophyll content of the control and M1 plants was estimated at specific intervals (4, 8, and 12 weeks after sowing) from five randomly selected plants per plot (by inserting the measurement tool into the lower surface of the leaf and closing the head) using a SPAD meter (Minolta, Europe).

The numbers of days from sowing to 50% flowering and 50% podding among the control and test plots were also recorded and statistically compared. The harvested M2 seeds were evaluated in a biochemical analysis.

### 2.5. Biochemical Analysis Method

#### 2.5.1. Estimation of Fiber Content (%)

For estimation of seed fibre content, two grams of the powdered seed sample (control and M1 seeds) of each variety (SL744 and SL958) was introduced into separate beakers followed by addition of distilled water (100 mL) and 10% H_2_SO_4_ (20 mL). The beaker was kept on a hot plate for 30 min. Thereafter, the suspension was filtered and then heated again using 1.25% NaOH for 30 min, filtered, and rinsed with hot water. It was allowed to drain, and the residue was scraped into a pre-weighed crucible (W_1_). It was then put into a muffle furnace to dry for 2 h at 600 °C, kept in a desiccator to cool, and weighed as W_2_ [26]. The percentage fibre content was then calculated as shown below:

Calculation:


W_0_ = Weight of empty crucible

W_1_ = Weight of crucible + residue

W_2_ = Weight of empty crucible + weight of residue after drying

% Crude fibre = (W_1_ − W_2_)/(W_1_ − W_0_) × 100

#### 2.5.2. Determination of Protein Content (%)

Micro Kjeldahl method was used to analyse the protein content of the control and M1 seeds of each variety (SL744 and SL958), which involved three steps: digestion, distillation, and titration [26]. The % nitrogen (N) and crude protein content were calculated as mentioned below:

Calculation:

% Nitrogen (N) = (TV × NA × 0.014 × DF)/(volume of aliquot × sample weight) × 100

% Crude protein (g) = C.F × % N

where,

TV = Titre value
NA = Normality of acid
Sample weight = 2 g.
DF = Dilution factor
Vol. of aliquot = 10 mL
Conversion factor C.F = 6.25.

#### 2.5.3. Determination of Lipid Content (%)

Two grams of the powdered seed sample (control and M1 seeds) of each variety (SL744 and SL958) were placed in separate labelled thimbles, and their mouths were covered with cotton wool. N-hexane (200 mL) was then added into the 250 mL extractor flask. The covered porous thimbles were placed in a condenser, and the Soxhlet apparatus was assembled. Extraction was allowed for about 5–6 h. Thereafter, the extracted sample in the thimbles was transferred into a dried crucible. The weight of the crucible and extracted sample was taken as W_1_. The crucible containing the sample was oven-dried at 105–110 °C for 1 h and later cooled in a desiccator, and the weight was taken as W_2_ [26]. The % weight of lipid was calculated as mentioned below:

Calculation:


W_0_ = Weight of empty crucible

W_1_ = Weight of crucible + grounded extracted sample (oil) before

W_2_ = Weight of empty crucible + weight of extracted sample (oil) after drying

% weight of lipid = (W_2_ − W_1_)/(W_1_ − W_0_) × 100

### 2.6. Statistical Analysis

The experimental data were collected from 5 randomly selected plants in each of the experimental plots (Figure 1). A two-way ANOVA was used to analyse the data through SPSS software (version 22), and the means were separated using Duncan’s multiple range test (DMRT) at 5% level of significance.

## 3. Results

### 3.1. Effect of Chemical Mutagens on Seed Germination

Data were recorded at three different periods (4DAS, 8DAS, and 10DAS) for the ten different treatments using the two varieties of soybean (SL744 and SL958) (Figure 2A,B). The highest seed germination percentage (76.0% seedlings/plot) was recorded in SL958 using 0.4% SA. The lowest value (30.33% seedlings/plot) was observed in SL958 using 0.6% MMS. The untreated control and treatment had same value (76% seedlings/plot) for SL958. However, in SL744, the % seed germination was higher (65.0% seedlings/plot) with 0.4% SA, and the lowest value (39.33.0% seedlings/plot) was observed in SL744 with 0.2% MMS compared to that of the control (53.0%).

### 3.2. Effects of Chemical Mutagens on Plant Height

The Table 1 shows the results of the effect of chemical mutagens on the plant heights of the control and M1 plants. The treatments differed significantly from each other and the untreated controls (*p* = 5%) in most of the data-recording periods. At 12 WAS, the T_9_-treated (0.4% SA) plots exhibited the highest plant height (37.84 ± 1.32 cm) for the SL958 variety, followed by T_2_ (35.06 ± 1.42 cm) with 0.2% EMS. Meanwhile, the lowest height (21.90 ± 0.63 cm) was recorded for T_7_ (0.6% MMS). Moreover, for the SL744 variety at 12 WAS, the T_2_ (0.2% EMS)-treated plots exhibited the highest plant height (24.80 ± 1.07 cm), and the lowest height was recorded for T_10_ (20.58 ± 0.30 cm) with 0.6% SA. These results, however, differed significantly from those of the untreated control plants (SL958: 26.09 ± 0.62 cm and SL744: 27.48 ± 0.74 cm).

### 3.3. Effect of Chemical Mutagens on Leaf Count

Data of leaf count were also collected from the same experimental plots to compare the effect of the three different chemical mutagens on the two soybean (SL744 and SL958) varieties, as shown in Table 2. The results showed that at four weeks after sowing (4 WAS), there were significant differences in the number of leaves among the treatments. This indicated that the chemical mutagens had a strong effect on the leaf count. However, at eight and twelve weeks after sowing (8 and 12 WAS), there was a highly significant difference in the number of leaves among the treatments, indicating that the treatments had a positive impact on the leaf count. At 4 WAS, the T7 treatment (0.6% MMS) caused a significant reduction in the number of leaves (17.47 ± 0.70 leaves/plant) in SL958, which was significantly different from the other treatments. At 8 and 12 WAS, the same treatment (0.6% MMS) exhibited the most significant reduction in the number of leaves (81.58 ± 4.03 leaves/plant and 87.00 ± 0.82 leaves/plant, respectively). These results differed significantly from those of all the treatments, including the untreated control plants (156.77 ± 1.05 leaves/plant and 160.73 ± 1.05 leaves/plant). At 12 WAS, the T9 treatment (0.4% SA) recorded the highest leaf count (234.33 ± 3.09 leaves/plant) in SL958. Similarly, in SL744, the highest leaf count (199.33 ± 2.62 leaves/plant) was observed in response to the T9 treatment (0.4% SA), which significantly differed from that of the untreated control plants (180.00 ± 1.63 and 160.73 ± 1.05).

### 3.4. Effect of Chemical Mutagens on Leaf Variation

**Figure 3:**
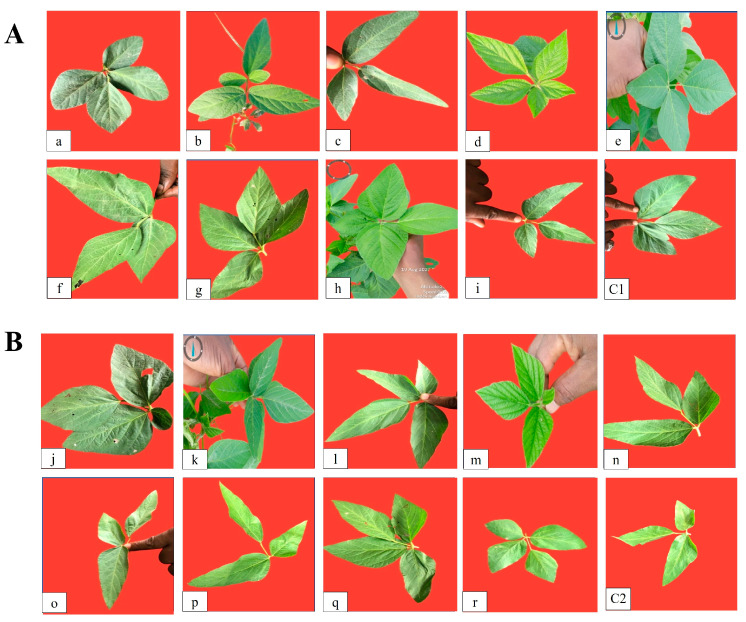
(**A**) Effect of different concentrations of three mutagens on the morphology of soybean (*Glycine max* L.) variety SL958 at 12 WAS. Mutagen I: Ethyl methane sulfonate (**a**) 0.2%, (**b**) 0.4%, (**c**) 0.6%. Mutagen II: Methyl methane sulfonate (**d**) 0.2%, (**e**) 0.4%, (**f**) 0.6%. Mutagen III: Sodium azide (**g**) 0.2%, (**h**) 0.4%, (**i**) 0.6%. (**C1**) Control: SL958 (without mutagen treatment). (**B**) Effect of different concentrations of three mutagens on the morphology of SL744 soybean (*Glycine max* L.) variety at 12 WAS. Mutagen I: Ethyl methane sulfonate (**j**) 0.2%, (**k**) 0.4%, (**l**) 0.6%. Mutagen II: Methyl methane sulfonate (**m**) 0.2%, (**n**) 0.4%, (**o**) 0.6%. Mutagen III: Sodium azide (**p**) 0.2%, (**q**) 0.4%, (**r**) 0.6%. (**C2**) Control: SL744 (without mutagen treatment).

Plant morphology is considered to be an important tool for the screening of desirable mutants. In our study, chemical mutagens caused the appearance of leaf abnormalities, including unifoliate, bifoliate, tetrafoliate, and pentafoliate characteristics (Figure 3A,B). Morphological changes, such as variations in leaf shape (broad and narrow leaves), were also observed in this study. Broad leaves were observed in the SL958 variety for 0.2% EMS (A: tetrafoliate), 0.2% MMS (G: tetrafoliate), 0.2% SA (D: pentafoliate), and 0.4% SA (H: tetrafoliate), while in the SL744 variety, broad leaves were observed for 0.2% EMS (J: tetrafoliate), 0.4% EMS (K: tetrafoliate), 0.6% MMS (O: trifoliate), 0.4% MMS (N: trifoliate), and 0.4% SA (Q: tetrafoliate).

Narrow leaves with pointed tips along with thinner stems were observed in SL958 for 0.4% EMS (B: pentafoliate), 0.6% EMS (C: trifoliate), 0.4% MMS (E: trifoliate), 0.6% MMS F: trifoliate), and 0.6% SA (I: trifoliate), in comparison to the untreated control plants. Meanwhile, in SL744, narrow leaves were observed for 0.6% EMS (L: tetrafoliate), 0.2% MMS (M: tetrafoliate), 0.2% SA (P: trifoliate), and 0.6% SA (R: tetrafoliate). It was interesting to note that in the pentafoliate leaves, the leaflets were much more narrow and the area was much more reduced than in their tetrafoliate counterparts, which had a broad leaf base.

### 3.5. Effect of Chemical Mutagens on Leaf Area (cm^2^)

Table 3 shows the results of the effect of the chemical mutagens on the leaf area of the control and M1 plants. The results revealed that at 4 WAS, the T_9_ plots (0.4% SA) recorded the highest leaf area (62.5 ± 0.82 cm^2^) in SL958, while the T_7_ plots (0.6% MMS) recorded the lowest (22.2 ± 0.00 cm^2^). Similarly, at 4 WAS, the T_9_ plots (0.4% SA) recorded the highest leaf area (61.06 ± 0.47 cm^2^) in SL744, while the T_4_ plots (0.6% EMS) recorded the lowest (22.72 ± 0.00 cm^2^).

However, at 8 WAS, the response in the T_2_ (0.2% EMS) plots differed significantly (*p* < 5%) from the untreated plots in having the highest leaf area (2628.4 ± 1.16 cm^2^) in SL958, while the T_8_ (0.2% SA) plots had the lowest (148.9 ± 0.50 cm^2^). In SL744, the T_5_ (0.2% MMS) plots recorded the highest leaf area (2042.6 ± 1.20), whereas the T_4_ plots (0.6% EMS) recorded the lowest (496.0 ± 1.08 cm^2^) leaf area. At 12 WAS, the results followed a similar trend. The T_9_ plot (0.4% SA) exhibited the highest leaf area (3625.8 ± 1.43 cm^2^) in SL958, while the T_7_ (0.6% MMS) plot recorded the lowest area (267.9 ± 1.09 cm^2^). In SL744, the T_9_ (0.4% SA) plots recorded the highest leaf area (2311.03 ± 3.65 cm^2^), whereas the T_10_ plots (0.6% SA) recorded the lowest (731.54 ± 1.11 cm^2^) leaf area compared to that of the untreated control (2190.6 ± 2.05 and 2256.6 ± 1.72).

### 3.6. Effect of Chemical Mutagens on Number of Days to 50% Flowering

The time (days) to reach 50% flowering for the untreated control and M1 plants is displayed in Table 4. In SL744 and SL958, the untreated control plants took the most number of days to reach 50% flowering (68.00 and 63.33 days, respectively), while the T_9_ (0.4% SA)-treated plants in SL958 took the least amount of time (39.67 ± 0.94 days). However, the T_7_-treated (0.6% MMS) M1 plants took the most number of days (50.33 ± 1.25 days) for the SL958 variety. Meanwhile, for SL744, T_8_ (0.2% SA) took the most number of days to reach 50% flowering (51.33 ± 0.47), while the T_9_ (0.4% SA)-treated plants for SL744 took the least amount of time (46.67 ± 1.70 days).

This revealed that the chemical mutagens at a low dose accelerated flowering in the M1 plants as compared to the untreated controls.

### 3.7. Effect of Chemical Mutagens on Podding

The time (days) to reach 50% podding in the untreated control and M1 plants is displayed in Table 5. It shows that the T_9_ (0.4% SA)-treated M1 plants of the SL958 variety exhibited early podding (69.00 ± 0.82 days) as compared to the untreated control plants (82.67 ± 1.25 days).

Similar results were observed for the T_9_ M1 plants of the SL744 variety, for which podding occurred at 77.00 ± 2.87 days, compared to the delayed podding of the untreated control plants (86.33 ± 0.94 days).

In terms of yield among both of the varieties, the highest yield (277.55 ± 1.37 pods/plant) was recorded for the M1 plants of the SL958 variety treated with 0.4% SA (T_9_), while the lowest (217.55 ± 2.20 pods/plant) was recorded for the T_4_ plants treated with 0.4% EMS compared to the untreated control (229.86 ± 0.96 pods/plant). However, in the SL744 variety, the highest yield (273.00 ± 4.55 pods/plant) was recorded for T_4_ (0.4% EMS), while the lowest (112.2 pods/plant) was observed in the SL744 variety treated with 0.2% EMS (T_2_). The results indicate that 0.4% SA effectively enhanced the yield of the SL958 variety.

### 3.8. Effects of Chemical Mutagens on Plant Chlorophyll Content (nmol/cm^2^)

Table 6 shows the effects of chemical mutagens on plant chlorophyll content at 4, 8, and 12 WAS. At 4 WAS, there was significant difference in chlorophyll content in the M1 plants of the SL744 variety, while for the SL958 variety, there was no significant difference among the T_2_ and T_3_; T_6_, T_5_, and T_7_; and T_4_, T_8_, T_9_, and T_10_ plots.

At 8 WAS, the chlorophyll content was found to be the highest (47.25 ± 3.07 nmol/cm^2^) in the T_2_ M1 plants (0.2% EMS) and the lowest (41.06 ± 4.06 nmol/cm^2^) in the T_10_ (0.6% SA) M1 plants of the SL744 variety. Meanwhile, in SL958, the highest chlorophyll content (45.76 ± 1.02 nmol/cm^2^) was found in the T9 plot and the lowest (40.97 ± 0.51) in the T_10_ plot.

However, at 12 WAS, among both of the varieties, the chlorophyll content was the highest (48.10 ± 0.71 nmol/cm^2^) in the T_2_ (0.2% EMS) plot of the SL958 variety and the lowest (41.33 ± 3.63 nmol/cm^2^) in the T_7_ (0.6% MMS) plot of the SL958 variety, which differed substantially from the control plants (39.54 nmol/cm^2^).

### 3.9. Effect of Chemical Mutagens on Protein, Lipid, and Fiber Content in M2 Seeds

Table 7 presents the percentages of protein, lipid, and fibre in the untreated control and M2 seeds. The results show that the T_9_ treatment (0.4% SA) recorded the highest protein percentage (47.04 ± 0.87%) in SL958 and lowest was recorded for the T_7_ treatment (0.6% MMS) (37.13 ± 0.0.72%) compared to the untreated control (39.87 ± 0.96%). However, in the SL744 variety, the highest (46.14 ± 0.64) was recorded in T_9_ (0.4% SA) and the lowest in T_10_ (0.6% SA) (39.82 ± 1.07%). This was significantly different compared to the untreated controls (36.64 ± 3.83%).

The highest lipid percentage (22.34 ± 0.59%) was observed in the T_9_ (0.4% SA) M2 plants of the SL958 variety, while the lowest (19.87 ± 0.47%) was recorded in the T_10_ plants (0.6% SA), as compared to the untreated controls (20.74 ± 0.64%). The highest lipid percentage (21.74 ^a^ ± 0.77%) was observed in the T_8_ (0.2% SA) M2 plants of the SL744 variety, while the lowest (18.69 ± 0.55%) was recorded in the T_7_ plants (0.6% MMS) compared to the untreated controls (21.39 ± 0.37%).

The highest fibre content was observed in the T_9_ plants (0.4% SA), which was 18.23 ± 0.12% in the M2 seeds of SL958, while the lowest (16.59 ^c^ ± 0.36%) was observed in the T_7_ plants (0.6% MMS) in SL958 compared to the controls (17.71 ± 0.39%). The highest fibre content was observed in the T_8_ plants (0.4% SA), which was 17.89 ± 0.39% in the M2 seeds of SL744, while the lowest (16.23 ± 0.93%) was observed in the T_6_ (0.4% MMS) plants in SL744 compared to the controls (16.99%).

## 4. Discussion

The present findings signify that induced mutagenesis can play a crucial role in improving the genetic makeup of crop plants in order to feed the teaming population of billions of people. Chemical mutagens caused the appearance of leaf variations including unifoliate, bifoliate, tetrafoliate, and pentafoliate characteristics. In our study, the seed germination percentage increased with lower doses of SA, EMS, and MMS (0.4%) and decreased with increasing dose (0.6%) compared to the untreated controls. Every living cell requires energy in the form of ATP molecules to carry out all biological reactions. When ATP levels are low, the rate of biological reactions inside the cell decreases [27]. Higher doses of SA (0.6%) can hinder ATP biosynthesis, resulting in a decreased availability of ATP molecules; as a result, the germination rate may slow down. Our findings are in consonance with those of Apparao (2005) [28], who reported that there was a reduction in germination percentage with higher doses of SA (25%) and an increase in germination with 5% SA in cowpea seeds. The decrease in seed germination count was observed in sesame seed with higher doses of SA (0.0776%) and an increased germination count was observed with lower doses (0.0473%) [29]. Furthermore, Aliyu et al. [30] reported that a nonlinear decline in mutagenic efficiency in *Sesamum indicum* was observed with increasing concentration and dose of sodium azide, and the best growth was recorded in terms of morphological and yield traits at a lower (0.5 mM) concentration of sodium azide. The decline in mutagenic effectiveness with increasing concentration of sodium azide might be attributed to increasing chromosome damage with increasing concentration of the mutagen.

Increased plant height was observed with lower doses (0.4%) of SA (37.8 cm) in the SL958 soybean variety and also with lower doses (0.2%) of EMS (24.80 cm) in the SL744 variety. This might be attributed to the effect of chemical mutagens on plant growth hormones. An increase in mutagen concentration often results in more physiological damage and direct DNA damage, as well as biochemical disturbances, auxin destruction, and changes in ascorbic acid content. These damages are phenotypically reflected in plant growth. The reduction in plant height caused by chemical mutagens can be attributed to any of the aforementioned factors. Our results are in agreement with the findings of Markeen et al. [31] in ‘urd bean’, who reported a decrease in plant height with higher doses of EMS (12%), attributed to gross injury at the cellular level. Our findings are also in consonance with the observations of OlaOlorun et al. [32]. In wheat plants, their results indicated that the plant height increased by 21.2 cm with 0.1% EMS and decreased by 16.3 cm with 0.7% EMS [32].

There was an increase in leaf number (229/234 leaves per plant) at a low dose of EMS (0.2%) and SA (0.4%), respectively, and a decrease in leaf number (164/220 leaves/plant) with high doses of EMS (0.6%) and SA (0.6%) in the M1 plants of the SL958 variety. Meanwhile, in the SL744 variety, the leaf number (199.33 leaves per plant) was higher with lower doses of SA (0.4%), and there was a decrease in leaf number (135.53 leaves/plant) with high doses of EMS (0.6%). Our observations are in consonance with the findings of Gupta et al. [33], who reported an increase in leaf number in soybean plants with lower doses of EMS (0.4%) and decrease in leaf number with higher doses of EMS (6%). However, it was also reported by Habib et al. [34] that there was a gradual reduction in the morphological characteristics of sunflower seedlings with the increasing dose of EMS in comparison to non-treated control seedlings. 

In our findings, the highest leaf area (3625.8 cm^2^) was observed in response to T_9_ (0.4% SA) in SL958, and the leaf area decreased (2703.4 cm^2^) with higher doses (0.6% SA). However, in the SL744 variety, the leaf area (2311.03 cm^2^) was higher with lower doses of SA (0.4%), and there was a decrease in leaf number (731.54 leaves/plant) with high doses of SA (0.6%). This result is similar to the findings of Al-Qurainy. Al-Qurainy reported that in *Eruca sativa* plants, with lower doses of SA (3%), the leaf area increases, and with higher doses (5%), the leaf area decreases [35]. Jabeen and Mirza found that in *Capsicum annum*, the leaf area increases with lower EMS doses (0.01%) and declines with higher doses (0.5%) [25].

In our study, broad leaves were observed with lower doses (0.2% SA pentafoliate leaves and 0.4% SA tetrafoliate leaves) in SL958, and narrow leaves were observed with higher doses (0.6%). Meanwhile, in SL744, broad leaves were observed with lower doses (0.2% EMS tetrafoliate leaves and 0.6% EMS tetrafoliate leaves), and narrow leaves were observed with higher doses (0.6%) of EMS. This is in consonance with the findings of Khursheed et al. [36], who reported that with lower doses of EMS (0.23%), broad leaves were observed, and with higher doses of EMS (0.25%), narrow leaves were observed in faba bean plants. It was also reported that with a much higher dose of EMS (0.3%), trifoliate leaves were observed, which is similar to our findings (0.6% EMS: trifoliate leaves). Ahire et al. [37] reported that there was an increased number of pentafoliate leaves (3) with lower doses EMS (0.2%) and decreased number of pentafoliate leaves (1) with higher doses (0.4%). No tetrafoliate leaves were observed with either of the doses (2% EMS or 0.4% EMS) in soybean plants. Espina et al. [38] reported that with higher doses of EMS (0.23%), tetrafoliate leaves were observed, and with lower doses of EMS (0.14%), pentafoliate leaves were observed in soybean plants [38].

In our study, the number of days to 50% flowering increased with increasing dose and decreased with decreasing dose of the chemicals. The T_9_ (0.4% SA)-treated plants in SL958 took the least time to flower (39.67 days) and the T_4_-treated plants (0.6% EMS) took the most time (51 days) in the SL958 variety. However, in the SL744 variety (51.33 days), the time was higher with lower doses of EMS (0.2%), and there was a decrease in the number of days (46.67 days) with the doses of SA (0.4%). Our results are in consonance with those of Kumar and Pandey (2019), who investigated the impact of EMS on *Coriandrum sativum* L. seeds by exposing them to various concentrations (0.1%, 0.3%, and 0.5%) for 3 and 5 h, respectively. They found that as EMS concentrations increased, there was a delay in the amount of time to 50% flowering compared to a control [39]. Samadi et al. (2022), while working with corn, reported a similar observation [40].

The plant chlorophyll content was observed to be higher (48.10 nmol/cm^2^) with lower doses of EMS (0.2%) and decreased (41.76 nmol/cm^2^) with increasing doses of MMS (0.6%) in the SL958 variety. Furthermore, in the SL744 variety, the chlorophyll content was observed to be higher (46.43 nmol/cm^2^) with lower doses of EMS (0.2%) and decreased (41.47 nmol/cm^2^) with increasing doses of SA (0.6%). Devi et al. [17] reported that there was a progressive increase in chlorophyll content with lower doses of 10% EMS and 10% SA and a decrease in chlorophyll content with higher doses of EMS (40%) and SA (40%) in chili (*Capsicum annuum* L.). Dinkar et al. [41] reported a higher frequency of chlorophyll mutations in chickpeas treated with 0.5% EMS. Selvaraj et al. [42] reported that lower doses of SA (5%) increase the chlorophyll content and higher doses (10%) decrease the chlorophyll content in rice plants.

Podding was observed to be higher (277.55 pods/plant) with 0.4% SA (SL958) and lower (112.22 pods/plant) with 0.2% EMS (SL744). Our results are contrary to the findings of Parchin et al. [43], who reported that a higher yield was obtained from fenugreek plants (*Trigonella foenum-graecum*) that received a higher dose of EMS (0.5%) and a lower yield was obtained with a lower dose of EMS (0.1%). Khan et al. [44] reported that high doses of SA (2.5%) decreased the yield and lower doses of SA (2%) increased the yield of barley plants. According to Etther et al. [45], *Cajanus cajan* plants treated with EMS had an increased grain yield per plant and 100-seed weight, which might be as a result of a high amount of mutagenic frequency, mutagenic effectiveness, and mutagenic efficiency at low concentrations of the mutagen.

In our study, the protein content of the M2 seeds of SL958 increased (47.04% TSP) with the decreased dose of SA (0.4%) and decreased (40.62% TSP) with the increased dose (0.6% SA). Jephter et al. [11] reported that there was an increase in the percentage of protein with lower doses of EMS (0.1%) and a decrease with higher doses of EMS (0.5%) in soybean seeds. The highest crude lipid content was observed (22.34%) with 0.4% SA, while lowest was recorded (19.87%) with 0.6% SA. Our findings are similar to that of Olorunmaiye et al. [46], who reported that high doses of EMS (1.2%) decreased the lipid content and lower doses (0.25%) increased the lipid content. In our study, the highest fibre content (18.23%) was observed with 0.4% SA, while the lowest (16.90%) was observed with 0.2% MMS. Olorunmaiye et al. [46] reported that high doses of EMS (1.25%) decreased the fibre content and lower doses (0.25%) increased the fibre content in groundnut seeds. Thus, the response to mutagens depends on the dose, plant type, and geographical location of the study. Therefore, the evaluation and elicitation of mutagens could be considered as an alternative method for the production of low-cost functional foods to diversify the soybean market. Moreover, it is important to identify elicitors that stimulate sprout growth and yield so as to improve plants’ nutritional properties.

## 5. Conclusions

The results obtained in the present study with regard to the application of chemical mutagens on soybean varieties have shown it is a simple and inexpensive method for improving agronomic characteristics. Their mutagenic effects manifest shortly after sowing seeds and are observable. Thus, chemical mutagens have been used in various crops to improve their yield and quality traits. The findings of this research have shown positive effects of SA treatment in SL958, which proved to be efficient in generating the highest leaf area (the tetrafoliate leaves were broad, but the pentafoliate leaves were narrow), and the highest number of pods/plants and protein content were recorded for the M1 plants. Furthermore, SL744 showed positive effects from the EMS treatment in terms of yield and quality traits, which indicates the potential of EMS and SA to induce useful mutations in soybean. The potential mutants generated should be selected for further evaluation (for assessing the transfer of trait(s) in subsequent generations) in a soybean breeding programme.

## Figures and Tables

**Table 1:** Effect of chemical mutagens on plant height (cm).

Treatments	Data Collection Periods					
	4 WAS		8 WAS		12 WAS	
	SL744	SL958	SL744	SL958	SL744	SL958
T_1_ (Control)	13.29 ± 0.68 ^cd^	9.29 ± 1.01 ^a^	26.13 ± 0.84 ^a^	21.99 ± 1.42 ^d^	27.48 ± 0.74 ^a^	26.09 ± 0.62 ^e^
T_2_	14.13 ± 0.96 ^a^	15.19 ± 0.89 ^a^	25.37 ± 0.52 ^a^	29.33 ± 0.63 ^b^	24.80 ± 1.07 ^b^	35.06 ± 1.42 ^b^
T_3_	12.10 ± 0.80 ^bc^	11.87 ± 1.12 ^bc^	21.29 ± 0.57 ^bc^	22.06 ± 0.23 ^d^	21.82 ± 1.24 ^cd^	24.27 ^ef^ ± 0.73
T_4_	10.13 ± 0.54 ^d^	9.75 ± 0.82 ^d^	20.87 ± 0.78 ^c^	17.43 ± 1.09 ^e^	24.17 ± 0.87 ^b^	22.44 ± 0.51 ^f^
T_5_	14.26 ± 1.15 ^a^	14.65 ± 0.81 ^a^	22.60 ± 0.93 ^b^	26.34 ± 0.90 ^c^	23.02 ± 0.82 ^bc^	28.40 ± 0.95 ^cd^
T_6_	9.99 ± 0.21 ^d^	9.99 ± 0.21 ^cd^	20.97 ± 0.67 ^c^	21.22 ± 0.86 ^d^	22.21 ± 1.03 ^cd^	25.53 ± 1.71 ^e^
T_7_	9.39 ± 0.82 ^d^	9.73 ± 0.64 ^d^	17.99 ± 0.74 ^d^	15.97 ± 1.00 ^e^	21.77 ± 0.17 ^cd^	21.90 ± 0.63 ^f^
T_8_	13.96 ± 0.64 ^a^	13.49 ± 0.90 ^ab^	17.95 ± 0.68 ^d^	25.37 ± 1.07 ^c^	21.31 ± 0.63 ^cd^	30.54 ± 2.37 ^c^
T_9_	14.19 ± 0.94 ^a^	14.25 ± 0.93 ^a^	19.19 ± 0.74 ^d^	36.35 ± 1.5 ^a^	23.18 ± 0.61 ^bc^	37.84 ± 1.32 ^a^
T_10_	11.09 ± 0.83 ^d^	10.12 ± 0.99 ^cd^	17.97 ± 0.69 ^d^	28.50 ± 0.91 ^b^	20.58 ± 0.30 ^d^	28.71 ± 0.92 ^cd^

The same letters in the same column are not significantly different from each other (*p* < 0.05). SD = Standard deviation ±, WAS = Weeks after sowing, T = Treatment (T_1_ = untreated control, T_2_ = 0.2% EMS, T_3_ = 0.4% EMS, T_4_ = 0.6% EMS, T_5_ = 0.2% MMS, T_6_ = 0.4% MMS, T_7_ = 0.6% MMS, T_8_ = 0.2% SA, T_9_ = 0.4% SA, T_10_ = 0.6% SA).

**Table 2:** Effect of chemical mutagens on the number of leaves per plant.

Treatments	Data Collection Periods					
	4 WAS		8 WAS		12 WAS	
	SL744	SL958	SL744	SL958	SL744	SL958
T_1_ (Control)	25.40 ± 0.0 ^bc^	26.47 ± 0.98 ^d^	147.33 ± 1.25 ^b^	156.77 ± 1.05 ^bc^	180.00 ± 1.63 ^c^	160.73 ± 1.05 ^d^
T_2_	23.13 ± 0.84 ^cd^	34.44 ± 0.97 ^a^	133.00 ± 2.16 ^b^	217.80 ± 5.37 ^a^	179.33 ± 2.49 ^c^	229.00 ± 5.35 ^ab^
T_3_	22.73 ± 0.38 ^cd^	24.78 ± 1.03 ^de^	126.00 ± 1.63 ^b^	155.87 ± 5.56 ^bc^	168.33 ± 4.99 ^c^	165.13 ± 2.11 ^d^
T_4_	22.40 ± 0.43 ^cd^	24.18 ± 0.95 ^f^	77.87 ± 1.33 ^b^	152.20 ± 5.37 ^c^	135.53 ± 5.72 ^g^	164.80 ± 2.47 ^d^
T_5_	31.27 ± 1.16 ^a^	28.51 ± 1.25 ^c^	178.80 ± 3.96 ^b^	218.00 ± 1.63 ^a^	190.33 ± 2.49 ^b^	227.33 ± 2.05 ^b^
T_6_	28.33 ± 1.25 ^ab^	22.20 ± 0.59 ^f^	134.00 ± 0.82 ^b^	215.67 ± 3.68 ^a^	179.80 ± 3.84 ^c^	220.07 ± 2.37 ^c^
T_7_	19.20 ± 1.07 ^d^	17.47 ± 0.70 ^g^	120.33 ± 1.25 ^b^	81.58 ± 4.03 ^d^	156.53 ± 3.13 ^e^	87.00 ± 0.82 ^f^
T_8_	25.67 ± 4.78 ^bc^	25.17 ± 0.94 ^de^	120.67 ± 1.70 ^b^	102.60 ± 6.25 ^d^	142.07 ± 2.25 ^fg^	146.00 ± 2.16 ^e^
T_9_	30.53 ± 1.84 ^a^	32.80 ± 0.75 ^ab^	200.00 ± 2.62 ^a^	193.87 ± 2.81 ^bc^	199.33 ± 2.62 ^a^	234.33 ± 3.09 ^a^
T_10_	28.20 ± 0.86 ^bc^	31.27 ± 0.99 ^b^	129.07 ± 11.44 ^b^	181.00 ± 1.63 ^b^	147.67 ± 0.05 ^fg^	220.33 ± 1.25 ^c^

The common letters in the same column are not significantly different from each other (*p* < 0.05). SD = Standard deviation ±, WAS = Weeks after sowing, T = Treatment (T_1_ = untreated control, T_2_ = 0.2% EMS, T_3_ = 0.4% EMS, T_4_ = 0.6% EMS, T_5_ = 0.2% MMS, T_6_ = 0.4% MMS, T_7_ = 0.6% MMS, T_8_ = 0.2% SA, T_9_ = 0.4% SA, T_10_ = 0.6% SA).

**Table 3:** Effect of chemical mutagens on leaf area (cm^2^) per plant.

Treatments	Data Collection Period					
	4 WAS		8 WAS		12 WAS	
	SL744	SL958	SL744	SL958	SL744	SL958
T_1_ (Control)	38.1 ± 0.41 ^b^	35.2 ± 0.47 ^c^	2111.2 ± 1.25 ^a^	2226.1 ± 1.07 ^b^	2190.6 ± 2.05 ^ab^	2256.6 ± 1.72 ^bc^
T_2_	59.80 ± 0.47 ^ab^	45.8 ± 0.47 ^b^	1605.3 ± 1.92 ^bc^	2628.4 ± 1.16 ^a^	1738.8 ± 1.25 ^b^	2790.6 ± 1.55 ^ab^
T_3_	38.62 ± 0.47 ^ab^	41.3 ± 0.47 ^ab^	1411.2 ± 2.18 ^c^	2069.1 ± 1.28 ^cd^	1527.4 ± 1.03 ^b^	2557.9 ± 1.51 ^b^
T_4_	22.72 ± 0.00 ^b^	32.1 ± 0.47 ^cd^	496.0 ± 1.08 ^ef^	1578.9 ± 0.82 ^c^	12,854.6 ± 1.5 ^b^	1865.9 ± 0.86 ^c^
T_5_	52.22 ± 0.47 ^ab^	57.2 ± 0.00 ^ab^	2042.6 ± 1.20 ^ab^	1018.6 ± 0.81 ^e^	2221.1 ± 1.27 ^ab^	2440.8 ± 0.86 ^c^
T_6_	32.06 ± 0.00 ^ab^	34.9 ± 0.82 ^cd^	1357.4 ± 1.95 ^cd^	239.2 ± 1.04 ^de^	1947.2 ± 1.58 ^b^	1091.1 ± 0.75 ^d^
T_7_	28.33 ± 0.82 ^b^	22.2 ± 0.00 ^e^	1070.9 ± 1.34 ^cd^	236.3 ± 1.70 ^bc^	939.1 ± 0.82 ^c^	267.9 ± 1.09 ^d^
T_8_	25.17 ± 0.00 ^b^	32.8 ± 0.82 ^cd^	520.0 ± 0.16 ^e^	148.9 ± 0.50 ^f^	1633.8 ± 2.03 ^b^	1946.1 ± 1.25 ^cd^
T_9_	61.06 ± 0.47 ^a^	62.5 ± 0.82 ^a^	1199.7 ± 1.02 ^cd^	2383.7 ± 2.56 ^ab^	2311.03 ± 3.65 ^a^	3625.8 ± 1.43 ^a^
T_10_	37.50 ± 0.40 ^b^	33.4 ± 0.67 ^d^	1987.4 ± 3.47 ^b^	998.2 ± 1.54 ^ef^	731.54 ± 1.11 ^c^	2703.4 ± 1.16 ^c^

The same letters in the same column are not significantly different from each other (*p* < 0.05). SD = Standard deviation ±, WAS = Weeks after sowing, T = Treatment (T_1_ = untreated control, T_2_ = 0.2% EMS, T_3_ = 0.4% EMS, T_4_ = 0.6% EMS, T_5_ = 0.2% MMS, T_6_ = 0.4% MMS, T_7_ = 0.6% MMS, T_8_ = 0.2% SA, T_9_ = 0.4% SA, T_10_ = 0.6% SA).

**Table 4:** Effect of chemical mutagens on flowering.

Treatments	Days to 50% Flowering	
	SL744	SL958
T_1_ (Control)	68.00 ± 0.82 ^a^	63.33 ± 1.25 ^a^
T_2_	47.67 ± 1.25 ^de^	43.00 ± 0.82 ^de^
T_3_	51.00 ± 1.63 ^bc^	46.00 ± 0.82 ^c^
T_4_	51.00 ± 0.83 ^bc^	45.00 ± 0.82 ^cd^
T_5_	47.00 ± 0.82 ^e^	48.33 ± 1.70 ^b^
T_6_	48.33 ± 1.70 ^cde^	49.00 ± 0.82 ^b^
T_7_	48.67 ± 1.25 ^bcde^	50.33 ± 1.25 ^b^
T_8_	51.33 ± 0.47 ^b^	41.33 ± 0.47 ^ef^
T_9_	46.67 ± 1.70 ^e^	39.67 ± 0.94 ^f^
T_10_	50.00 ± 0.82 ^bcd^	42.67 ± 0.47 ^e^

The same letters in the same column are not significantly different from each other (*p* < 0.05). SD = Standard deviation ±, WAS = Weeks after sowing, T = Treatment (T_1_ = untreated control, T_2_ = 0.2% EMS, T_3_ = 0.4% EMS, T_4_ = 0.6% EMS, T_5_ = 0.2% MMS, T_6_ = 0.4% MMS, T_7_ = 0.6% MMS, T_8_ = 0.2% SA, T_9_ = 0.4% SA, T_10_ = 0.6% SA).

**Table 5:** Effect of chemical mutagens on days to 50% podding and pod yield.

	Pod Yield		Days to 50% Podding	
Treatments	SL744	SL958	SL744	SL958
T_1_ (Control)	164.33 ± 8.58 ^d^	229.86 ± 0.96 ^e^	86.33 ± 0.94 ^a^	82.67 ± 1.25 ^a^
T_2_	112.22 ± 5.92 ^e^	228.44 ± 1.23 ^e^	80.33 ± 0.94 ^e^	70.67 ± 1.25 ^cd^
T_3_	264.11 ± 3.09 ^ab^	261.00 ± 0.82 ^b^	81.00 ± 0.47 ^cd^	74.67 ± 1.25 ^b^
T_4_	273.00 ± 4.55 ^a^	217.55 ± 2.20 ^g^	79.67 ± 1.41 ^a^	74.33 ± 1.25 ^b^
T_5_	260.44 ± 30.99 ^ab^	228.00 ± 2.67 ^d^	85.00 ± 0.47 ^a^	80.00 ± 0.82 ^a^
T_6_	232.22 ± 1.10 ^ab^	222.78 ± 0.82 ^e^	85.33 ± 3.56 ^bc^	80.67 ± 1.25 ^a^
T_7_	179.33 ± 33.07 ^cd^	270.11 ± 1.10 ^f^	84.33 ± 0.47 ^a^	72.33 ± 1.70 ^c^
T_8_	146.66 ± 50.87 ^bc^	269.78 ± 0.95 ^b^	85.33 ± 1.41 ^cd^	73.67 ± 2.16 ^d^
T_9_	206.54 ± 23.47 ^de^	277.55 ± 1.37 ^b^	77.00 ± 2.87 ^cd^	69.00 ± 0.82 ^a^
T_10_	216.67 ± 4.34 ^bc^	276.69 ± 1.23 ^a^	84.33 ± 0.47 ^bc^	73.67 ± 1.70 ^c^

The same in letters in the same column are not significantly different from each other (*p* < 0.05). SD = Standard deviation ±, WAS = Weeks after sowing, T = Treatment (T_1_ = untreated control, T_2_ = 0.2% EMS, T_3_ = 0.4% EMS, T_4_ = 0.6% EMS, T_5_ = 0.2% MMS, T_6_ = 0.4% MMS, T_7_ = 0.6% MMS, T_8_ = 0.2% SA, T_9_ = 0.4% SA, T_10_ = 0.6% SA).

**Table 6:** Effect of chemical mutagens on chlorophyll content (nmol/cm^2^).

Treatments	Data Collection Periods					
	4 WAS		8 WAS		12 WAS	
	SL744	SL958	SL744	SL958	SL744	SL958
T_1_ (Control)	34.58 ± 1.88 ^cd^	37.75 ± 4.29 ^a^	45.27 ± 1.61 ^a^	41.27 ± 0.55 ^b^	45.78 ± 2.10 ^a^	39.54 ± 0.55 ^d^
T_2_	42.09 ± 1.70 ^a^	39.61 ± 0.61 ^a^	47.25 ± 3.07 ^a^	44.21 ± 0.71 ^ab^	46.43 ± 2.50 ^a^	48.10 ± 0.71 ^abc^
T_3_	41.04 ± 0.87 ^ab^	38.71 ± 1.55 ^a^	45.04 ± 2.06 ^a^	44.09 ± 0.65 ^ab^	44.10 ± 1.63 ^a^	45.17 ± 0.65 ^a^
T_4_	38.06 ± 3.15 ^abc^	35.95 ± 2.30 ^ab^	43.57 ± 1.65 ^a^	42.80 ± 0.65 ^ab^	44.01 ± 3.37 ^a^	44.83 ± 0.65 ^ab^
T_5_	35.27 ± 2.28 ^cd^	37.79 ± 2.30 ^b^	45.51 ± 3.15 ^a^	44.66 ± 0.61 ^ab^	46.47 ± 3.08 ^a^	44.28 ± 0.61 ^abc^
T_6_	32.89 ± 2.65 ^cd^	34.89 ± 0.75 ^a^	43.56 ± 1.21 ^a^	42.97 ± 1.71 ^ab^	44.35 ± 7.18 ^a^	43.61 ± 1.72 ^abc^
T_7_	31.57 ± 1.14 ^d^	31.85 ± 1.07 ^b^	42.26 ± 6.32 ^a^	42.88 ± 3.63 ^ab^	44.32 ± 0.94 ^a^	41.33 ± 3.63 ^cd^
T_8_	35.02 ± 3.58 ^cd^	35.63 ± 1.45 ^ab^	42.83 ± 3.38 ^a^	43.19 ± 0.61 ^ab^	43.75 ± 4.22 ^a^	44.34 c ± 0.6 ^ab^
T_9_	35.95 ± 2.84 ^bcd^	38.77 ± 3.45 ^ab^	42.90 ± 1.48 ^a^	45.76 ± 1.02 ^a^	44.31 ± 0.74 ^a^	45.38 ± 1.02 ^a^
T_10_	32.87 ± 2.25 ^cd^	35.76 ± 3.19 ^ab^	41.06 ± 4.06 ^a^	40.97 ± 0.5 ^b^	41.47 ± 4.07 ^a^	41.76 ± 0.51 ^bcd^

The same letters in the same column are not significantly different from each other (*p* < 0.05). SD = Standard deviation ±, WAS = Weeks after sowing, T = Treatment (T_1_ = untreated control, T_2_ = 0.2% EMS, T_3_ = 0.4% EMS, T_4_ = 0.6% EMS, T_5_ = 0.2% MMS, T_6_ = 0.4% MMS, T_7_ = 0.6% MMS, T_8_ = 0.2% SA, T_9_ = 0.4% SA, T_10_ = 0.6% SA).

**Table 7:** Effect of chemical mutagens on protein, lipid, and fibre content (%) in M2 seeds.

Treatments	Protein		Lipid		Fiber	
	SL744	SL958	SL744	SL958	SL744	SL958
T_1_ (Control)	36.64 ± 3.83 ^c^	39.87 ± 0.96 ^c^	21.39 ± 0.37 ^ab^	20.74 ± 0.64 ^abc^	16.99 ± 0.30 ^a^	17.71 ± 0.39 ^ab^
T_2_	42.45 ± 0.25 ^b^	43.25 ± 0.42 ^b^	20.62 ± 0.41 ^bc^	21.20 ± 0.05 ^bc^	17.56 ± 0.27 ^a^	17.69 ± 0.26 ^ab^
T_3_	46.01 ± 0.51 ^a^	45.46 ± 0.19 ^a^	20.76 ± 0.14 ^bc^	21.67 ± 0.71 ^a^	16.54 ± 0.39 ^a^	17.24 ± 0.22 ^bc^
T_4_	41.36 ± 1.04 ^b^	40.34 ± 0.63 ^cd^	20.63 ± 0.31 ^bc^	21.68 ± 0.58 ^a^	16.28 ± 0.15 ^a^	17.14 ± 0.16 ^bc^
T_5_	41.72 ± 0.44 ^b^	40.91 ± 0.19 ^cd^	20.04 ± 1.09 ^bcd^	20.32 ± 0.41 ^bc^	16.32 ± 0.27 ^a^	16.90 ± 0.19 ^c^
T_6_	40.49 ± 0.48 ^b^	41.66 ± 0.47 ^c^	19.19 ± 0.44 ^de^	21.09 ± 0.88 ^abc^	16.23 ± 0.93 ^a^	16.73 ± 0.52 ^c^
T_7_	39.89 ± 0.72 ^b^	38.13 ± 0.81 ^e^	18.69 ± 0.55 ^e^	20.49 ± 0.11 ^abc^	16.28 ± 0.09 ^a^	16.59 ^c^ ± 0.36
T_8_	41.00 ± 0.47 ^b^	45.93 ± 0.99 ^a^	21.74 ± 0.77 ^a^	21.73 ± 0.38 ^a^	17.89 ± 0.39 ^a^	17.18 ± 0.03 ^bc^
T_9_	46.14 ± 0.64 ^a^	47.04 ± 0.87 ^a^	21.37 ± 0.50 ^ab^	22.34 ± 0.59 ^a^	17.43 ± 0.05 ^a^	18.23 ± 0.12 ^a^
T_10_	39.82 ± 1.07 ^b^	40.62 ± 0.41 ^cd^	19.95 ± 0.94 ^cde^	19.87 ± 0.47 ^c^	16.54 ± 0.53 ^a^	17.12 ± 0.23 ^bc^

The same letters in the same column are not significantly different from each other (*p* < 0.05). SD = Standard deviation ±, WAS = Weeks after sowing, T = Treatment (T_1_ = untreated control, T_2_ = 0.2% EMS, T_3_ = 0.4% EMS, T_4_ = 0.6% EMS, T_5_ = 0.2% MMS, T_6_ = 0.4% MMS, T_7_ = 0.6% MMS, T_8_ = 0.2% SA, T_9_ = 0.4% SA, T_10_ = 0.6% SA).

## Data Availability

Data will be made available on request.
